# Activation of the Hippo Pathway in *Rana sylvatica*: Yapping Stops in Response to Anoxia

**DOI:** 10.3390/life11121422

**Published:** 2021-12-17

**Authors:** Aakriti Gupta, Kenneth B. Storey

**Affiliations:** Institute of Biochemistry & Department of Biology, Carleton University, 1125 Colonel By Drive, Ottawa, ON K1S 5B6, Canada; aakritigupta@cmail.carleton.ca

**Keywords:** Hippo pathway, MST, SAV, LATS1/2, MOB, YAP, TAZ, TEAD, OCT4 and SOX2, anoxia, metabolic rate depression, energy stress

## Abstract

Wood frogs (*Rana sylvatica*) display well-developed anoxia tolerance as one component of their capacity to endure prolonged whole-body freezing during the winter months. Under anoxic conditions, multiple cellular responses are triggered to efficiently cope with stress by suppressing gene transcription and promoting activation of mechanisms that support cell survival. Activation of the Hippo signaling pathway initiates a cascade of protein kinase reactions that end with phosphorylation of YAP protein. Multiple pathway components of the Hippo pathway were analyzed via immunoblotting, qPCR or DNA-binding ELISAs to assess the effects of 24 h anoxia and 4 h aerobic recovery, compared with controls, on liver and heart metabolism of wood frogs. Immunoblot results showed significant increases in the relative levels of multiple proteins of the Hippo pathway representing an overall activation of the pathway in both organs under anoxia stress. Upregulation of transcript levels further confirmed this. A decrease in YAP and TEAD protein levels in the nuclear fraction also indicated reduced translocation of these proteins. Decreased DNA-binding activity of TEAD at the promoter region also suggested repression of gene transcription of its downstream targets such as SOX2 and OCT4. Furthermore, changes in the protein levels of two downstream targets of TEAD, OCT4 and SOX2, established regulated transcriptional activity and could possibly be associated with the activation of the Hippo pathway. Increased levels of TAZ in anoxic hearts also suggested its involvement in the repair mechanism for damage caused to cardiac muscles during anoxia. In summary, this study provides the first insights into the role of the Hippo pathway in maintaining cellular homeostasis in response to anoxia in amphibians.

## 1. Introduction

The freeze-tolerant wood frog (*Rana sylvatica*) displays an incredible survival strategy to endure the seasonal cold of winter. These frogs can survive the freezing of 65–70% of total body water that accumulates as ice in extracellular spaces. In the frozen state, frogs do not show any vital signs: no breathing, heartbeat, blood circulation, or measurable neural conductivity [1,2]. Loss of water into extracellular ice leads to a strong reduction in cell volume while also increasing cellular osmolality. High quantities of glucose are produced and packed into cells to act as a cryoprotectant to protect cells from damage. Glucose is produced from glycogen stored in the liver and is distributed to all other tissues when triggered by ice nucleation on the skin. As a result, tissue and plasma glucose concentrations increase from 1–5 mM (when unfrozen) to as high as 200–300 mM as frogs freeze [2,3]. Because freezing halts blood circulation, tissues rapidly become ischemic and anoxic [4,5]. Hence, wood frog survival of freezing also depends on a well-developed anoxia tolerance. Prolonged exposure to anoxia typically causes an imbalance in ATP production vs. utilization. ATP production decreases steadily when mitochondrial oxygen-based ATP synthesis is impaired since the ATP yield from anaerobic glycolysis is only a small fraction of that produced from aerobic respiration [6]. When oxygen-restricted by freezing, frogs switch to “survival mode” [7] which includes a transition to the use of fermentation fuels, activation of cell survival pathways, and suppression of ATP-expensive nonessential cellular processes [8]. One pathway that may be involved in regulating the transition into anaerobiosis in cells of anoxia-tolerant species is the Hippo or YAP signaling pathway. However, the potential involvement of this pathway in freeze tolerance has never before been considered.

**Figure 1:**
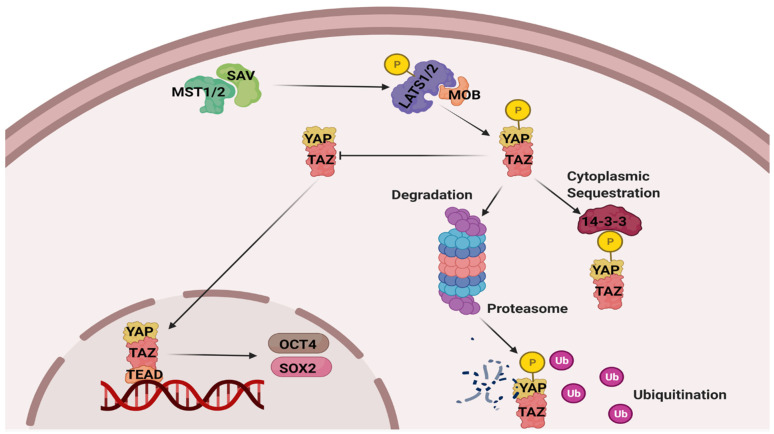
Schematic representation of the Hippo pathway. Upon encountering stress, the pathway gets activated and initiates a series of phosphorylation, where MST1/2 interacts with SAV to phosphorylate LATS1/2 that further phosphorylates YAP. Depending on the site of phosphorylation, either p-YAP interacts with 14-3-3 to sequester in cytoplasm or attach to proteosome to degrade via ubiquitination. Unphosphorylated YAP/TAZ translocates to the nucleus and forms complex with TEAD. The YAP/TAZ/TEAD complex binds to promoter region of DNA to initiate gene transcription and activation. MST: mammalian Ste20-like kinases ½, SAV: salvador 1, LATS1/2: large tumor suppressor 1 and 2 kinases, MOB: Mps one Binder, YAP: Yes-associated protein, TAZ: transcriptional coactivator with PDZ-binding motif, TEAD: TEA domain family member, OCT4: Octamer-binding transcription factor 4, and SOX2: SRY (sex determining region Y)-box 2. Credit “Created with BioRender.com” accessed on 14 October 2021.

The Hippo pathway is a signaling network that controls a variety of cell processes involved in cell proliferation, differentiation, and cell death. It is also known to regulate organ size by controlling both apoptosis and cell proliferation [9]. The pathway can be stimulated by multiple cellular stresses which include energy crisis, oxygen stress, reactive oxygen species, mechanical stress, and DNA damage [10]. Most of the pathway components are highly conserved, including in amphibians [11,12,13,14], and the mode of pathway activation depends on the stress signal involved. The pathway is activated when stress-specific signals lead to phosphorylation of mammalian Ste20-like kinases 1/2 (MST1/2) (Thr183 and Thr180), and MST1/2 binds to its regulatory subunit, salvador 1 (SAV1), to form an active protein that can phosphorylate large tumor suppressor 1 and 2 kinases (LATS1/2) on T1079 for LATS1 or T1041 for LATS2 [15]. Active LATS1/2 phosphorylates yes-associated protein (YAP) and the transcriptional coactivator with PDZ-binding motif (TAZ) at S127 and S381, respectively. This stabilizes a YAP/TAZ complex. Phosphorylation of YAP/TAZ sequesters the complex in the cytoplasm and leads to ubiquitination. By contrast, nonphosphorylated YAP/TAZ translocates to the nucleus and binds to the TEA domain family member (TEAD) protein to activate stress-dependent transcription factors. Amongst others, transcription factors activated by this pathway are OCT4 and SOX2 (Figure 1) [16,17]. In fact, YAP/TAZ is a part of the TSO (TEAD–SMAD–OCT4) complex and acts as a repressor by recruiting NuRD (a multicomponent chromatin remodeling complex that regulates gene transcription) [18], whereas when released from TSO, OCT4 acts as a stress-responsive transcription factor to regulate expression of genes involved in antioxidant defense [19,20,21].

Anoxic conditions can lead to cell quiescence (or even cell death), activating the Hippo pathway to result in phosphorylation of YAP/TAZ and inhibiting its nuclear localization [22,23,24,25]. This prevents activation of genes that might lead to apoptosis or other energy expensive processes [26]. The Hippo pathway also promotes cell survival. Stress responsive regulation of the Hippo pathway can lead either to activation of cell survival genes by translocation of unphosphorylated YAP/TAZ into the nucleus, or other stresses (e.g., low energy, membrane shear stress) can trigger phosphorylation of YAP to halt gene transcription [10,27,28]. Since anoxia tolerance is a crucial component of freezing survival for wood frogs, it is important to analyze the potential role played by the Hippo pathway with respect to winter cryopreservation in this amazing freeze-tolerant species.

## 2. Materials and Methods

### 2.1. Animal Treatment

Male wood frogs (weighing 5–7 g) were collected from breeding ponds near Ottawa, Ontario, Canada in early spring. Frogs were briefly washed in a tetracycline bath and then transferred to plastic containers lined with damp sphagnum moss, followed by acclimation at 4 °C for ~2 weeks. Control frogs were sampled from this condition. For anoxia exposure, frogs were treated as described by Gerber et al. [29]. Briefly, animals (4–5 per jar) were placed into plastic jars (sitting in ice) that were pre-flushed with nitrogen gas for 20 min and contained a pad of pre-wetted paper towels on the bottom (wetted with water previously bubbled with 100% nitrogen gas). Jars were again flushed with nitrogen gas before sealing both input and output vents present on the lids. The jars were returned to 5 °C for 24 h. After the anoxia treatment, frogs in half of the jars were sampled as the 24 h anoxic group, whereas the remaining frogs were transferred to other jars with normal air. These jars were returned to 5 °C for 4 h as an aerobic recovery period and then sampled. Frogs were euthanized by pithing and tissues were dissected rapidly for all three conditions (control, 24 h anoxia, 4 h recovery), immediately frozen in liquid nitrogen, and stored at −80 °C until use. All animal experiments followed the guidelines of the Canadian Council on Animal Care and had prior approval from the Carleton University Animal Care Committee (protocol no. 106935).

### 2.2. Total Protein Extractions for Immunoblots

Total protein was extracted from frozen heart and liver samples as described by Gerber et al. [29]. Briefly, frozen tissue samples (previously stored at −80 °C) were weighed and mixed 1:2 *w*/*v* with homogenization buffer (20 mM Hepes, pH 7.4, 100 mM NaCl, 0.1 mM EDTA, 10 mM NaF, 1 mM Na3VO4, and 10 mM β-glycerophosphate) with the immediate addition of 1 mM phenylmethylsulfonyl fluoride and 1 µL/mL protease inhibitor cocktail (BioShop, Burlington, ON, Canada, catalog no. PIC001.1) (reconstituted in 100 mL of deionized water). Samples were then immediately homogenized using a Polytron PT10 homogenizer and then stored on ice for ~15 min. Samples were centrifuged at 12,000× *g* for 15 min at 4 °C. The supernatant was collected, and the protein concentrations were determined using the Coomassie blue dye-binding method using the Bio-Rad prepared reagent (Bio-Rad Laboratories, Hercules, CA, USA; Cat # 500-0006). The concentrations of the samples were then standardized to 10 μg/μL by the addition of calculated small volumes of homogenization buffer. Standardized total protein extracts were then mixed 1:1 *v*/*v* with 2X SDS (sodium dodecyl sulphate) buffer (100 mM Tris-HCl, 20% *v*/*v* glycerol, 4% *w*/*v* SDS, 0.2% *w*/*v* bromophenol blue, and 10% *v*/*v* 2-mercaptoethanol) and then boiled for 5 min in a water bath followed by snap chilling on ice for 10 min and stored at −80 °C until further use.

### 2.3. Nuclear Protein Extractions for Immunoblots

Frozen samples of liver and heart were weighed and homogenized 1:5 *w*/*v* in buffer A (10 mM HEPES, pH 7.9; 10 mM KCl; 10 mM EDTA; 20 mM β-glycerophosphate) with addition of 10 μL of 100 mM dithiothreitol (DTT) and 10 μL of protease inhibitor cocktail added per mL. Tissue was disrupted using a Dounce homogenizer with 4–5 strokes. Samples were incubated on ice for 25 min and then centrifuged at 12,000× *g* for 15 min at 4 °C. Supernatant was transferred to pre-chilled new tubes and stored as the cytoplasmic fraction.

Pellets were re-suspended in 1:5 *w*/*v* (based on original sample weights) in homogenization buffer B (100 mM HEPES; 2 M NaCl; 5 mM EDTA; 50% *v*/*v* glycerol; 100 mM β-glycerol phosphate pH 7.9; 100 mM DTT and protease inhibitor cocktail at 1:1000). Samples were sonicated using a Polytron PT1000 homogenizer (Brinkmann Instruments, Rexdale, ON, Canada) for 5 s and then incubated on ice for 10 min followed by centrifugation at 14,000× *g* for 15 min at 4 °C. The supernatant was transferred to pre-chilled new tubes and stored as the nuclear fraction.

Protein concentrations in both cytoplasmic and nuclear extracts were measured using the Bio-Rad protein assay. Samples were checked for their integrity and the purity of both fractions by running two SDS-PAGE gels each with cytoplasmic and corresponding nuclear fractions. The membranes were probed with anti-histone H3 (Cell Signaling, Beverly, MA, USA, catalog no. 9715) and alpha-tubulin antibody (Santa Cruz Biotechnology, Santa Cruz, CA, USA. catalog no. sc-5286). Histone H3 served as the nuclear marker and alpha-tubulin as the cytoplasmic marker.

### 2.4. SDS PAGE and Western Blotting

The procedure described by Gerber et al., 2016 [29] was followed to run the samples on SDS PAGE gels. Briefly, 5% upper stacking gels and 10–15% resolving gels (depending on the molecular weight of the protein of interest) were used with 5 µg PiNK Plus Prestained Protein Ladder (Froggabio: PM005-0500) loaded in the first lane as a MW marker. Samples of 20 µg protein for all three conditions (control, 24 h anoxia, and 4 h recovery after anoxia, all *n* = 4) were loaded in other lanes. Gels were run at a constant voltage of 180 V until the band in the ladder lane (corresponding closest to the MW of the protein of interest) was well resolved from other bands. Proteins were then transferred from the gels to PVDF (polyvinylidene difluoride) membranes (Millipore, Etobicoke, ON, Canada, catalog no. IPVH07850, 45 μm pore) by electroblotting in 1X transfer buffer (25 mM Tris pH 8.5, 192 mM glycine, 20% methanol) at a constant current of 160 mA at 4 °C for 90 min. Subsequently, blots were blocked with 3% milk in TBST (20 mM Tris base, pH 7.6, 140 mM NaCl, 0.05% *v*/*v* Tween-20) for 30 min. Membranes were then washed for 3 × 5 min each followed by incubation with a specific primary antibody (diluted 1:1000 in TBST) overnight at 4 °C. Subsequently, the membranes were washed 3 × 5 min with TBST and incubated with horseradish peroxide-linked secondary antibody specific for the primary antibody for 30 min. Bands on membranes were visualized using hydrogen peroxide and luminol and quantified using a Chemi-Genius Bioimager (Syngene, Frederick, MD, USA).

The antibodies for OCT4 (Catalog no. GTX100468) and SOX2 (Catalog no. GTX101507) were purchased from GeneTex (Irvine, CA, USA). Antibodies for Mst-1 (Catalog no. A12963), Mst-2 (Catalog no. A6992), Sav-1 (Catalog no. A9980), LATS (Catalog no. A16249), p-LATS (Catalog no. AP0880), YAP (Catalog no. A1002), p-YAP (Catalog no. AP0489), TAZ (Catalog no. A12722), and TEAD1 (Catalog no. A6768) were purchased from Abclonal (Woburn, MA, USA). An anti-rabbit IgG conjugated with horseradish peroxidase (catalog no. APA007P.2, BioShop, Burlington, ON, Canada) was used as a secondary antibody.

### 2.5. Total Protein Extractions for TF ELISA

Total protein was extracted for all three conditions (control, 24 h anoxia, 4 h recovery) from frozen samples of heart and liver following the procedure described by Gupta and Storey, 2020 [20]. Briefly, samples were weighed and homogenized in a lysis buffer cocktail that included a protease inhibitor (BioShop, Burlington, ON, Canada, Catalog No. PIC001). Samples were incubated on ice for 30 min and later centrifuged at 14,000× *g* for 20 min at 4 °C. Supernatants were collected and protein concentrations were measured using Bio-Rad assay. Sample integrity was checked by running aliquots on SDS PAGE as described above.

### 2.6. DNA-Binding Activity Using TF ELISA

Biotin labeled DNA oligonucleotides corresponding to the binding site of TEAD were used to determine the binding capacity of the transcription factor to DNA. The following consensus sequences were used:

TEAD (5′Biotin-TGCCTAAATTTGGAATGTTCTGCT 3′)
TEAD complementary (5′ AGCAGAACATTCCAAATTTAGGCA 3′)

A standard protocol from previous work was followed [20]. The primary antibody for TEAD (described above) was used at 1:1000 *v*/*v* dilution in phosphate-buffered saline (PBST) containing 137 mM NaCl, 2.7 mM KCl, 10 mM Na2HPO4, 2 mM KH2PO4, pH 7.4) and 0.1% Tween-20.

### 2.7. RNA Isolation and cDNA Synthesis

RNA was isolated from frozen tissue samples of heart and liver following the method described by Gupta and Storey (2021) [30]. Briefly, samples were homogenized in TRIzol (BioShop, TRI118.100) at 1:20 *w*/*v* using a Polytron PT10 homogenizer and incubated at room temperature for 5 min. Subsequently, chloroform was added at 1:4 *w*/*v* and vortexed well. Samples were incubated for 5 min and then centrifuged at 10,000 rpm for 15 min at 4 °C. The upper aqueous layer was collected, and RNA was precipitated by incubating with 500 μL isopropanol at room temperature for 15 min. Samples were then centrifuged at 12,000 rpm for 15 min at 4 °C, and the supernatant was discarded. The pellet was washed with 70% ethanol and dissolved in autoclaved distilled water. The purity of RNA was checked by determining the OD 260/280 ratio and integrity was checked using agarose gel electrophoresis. Sample concentrations were standardized to 1 µg/µL. cDNA was synthesized from the RNA samples following the protocol of Gupta and Storey, 2021 [30], and cDNA was stored at −20 °C till further use.

### 2.8. Primer Design and qPCR

Forward and reverse primers were designed for mst1, mst2, sav, taz, tead, and yap. Since the genome of wood frogs is not sequenced, we identified consensus sequences by aligning sequences from several vertebrate species to identify conserved regions. Primer Blast on the NCBI was used to design primers from the conserved sequences. Primers used were:

mst1: Forward 5′ GTTGGGGCATGTGAGGGAGACT 3′
  Reverse 5′ CTCTGGCGGGCACAATGACAC 3′
mst2: Forward 5′ GAAGGGAAGCCGCCGTATGC 3′
  Reverse 5′ TGGGTGGTGGATTTGTGGGGA 3′
sav: Forward 5′ GAAAGAGACCTCCCCGCTGCT 3′
  Reverse 5′ TGGGCAGATATCAGTCCGTCTCG 3′
taz: Forward 5′ GGACACGCCGCTCATCACA 3′
  Reverse 5′ GTGCAAGTTCCACAGGTGCTTT 3′
tead: Forward 5′ CGTTTGGGAAACAAGTCGTGGAG 3′
  Reverse 5′ ACATCGGGGAGCGGTTTATCC 3′
yap: Forward 5′ TGCCCATGCGGATGAGGAAAC 3′
  Reverse 5′ GCTGATCCCCCATCTGTGCTG 3′
β-actin: Forward 5′-AGAAGTCGTGCCAGGCATCA-3′
  Reverse 5′-AGGAGGAAGCTATCCGTGTT-3′

The qPCR reaction was performed as described in previously [31,32] using a CFX-96 Real-Time PCR detection system (Bio-Rad, Hercules, CA, USA). The PCR reaction cycles used were as follows:

β-actin: 95 °C for 3 min and then 40 cycles of (95 °C 20 s, 53.8 °C 30 s, and 72 °C 20 s)
mst1, mst2, sav, taz, and tead: 95 °C 3 min and then 40 cycles of (95 °C 20 s, 54.3 °C 30 s, and 72 °C 20 s)
yap: 95 °C 3 min and then 40 cycles of (95 °C 10 s, 52.0 °C 30 s, and 72 °C 20 s)

PCR reaction runs were followed by a melt curve analysis to ensure a single product during amplification, and a final hold at 4 °C was applied. The primers were tested by performing a two-fold serial dilution curve test following MIQE guidelines [33] to ensure non-amplification primer dimers. Beta-actin was used as the reference gene for standardization since its expression did not change significantly between all three experimental conditions [34].

#### Statistical Analysis

The Chemi Genius Bioimager was used to image immunoblots, and band intensities were quantified using the associated Gene Tools software. Blots were then Coomassie-stained, and the summed intensity of the group of bands well separated from the band of interest was used to standardized protein band intensities to account for any minor differences that occurred during sample loading [35]. The results for qPCR were analyzed using the ∆∆Ct method [33,36]. Control values were set to 1, and the fold change for 24 h anoxia and 4 h recovery was calculated relative to control values to ease data interpretation. One-way ANOVA followed by a Tukey post hoc test (*n* = 4) was used to analyze the standardized values; the program RBioplot [37] was used and *p* < 0.05 was accepted as a significant difference between groups.

## 3. Results

### 3.1. Protein Levels of Cytoplasmic Components of the Hippo Pathway

**Figure 2:**
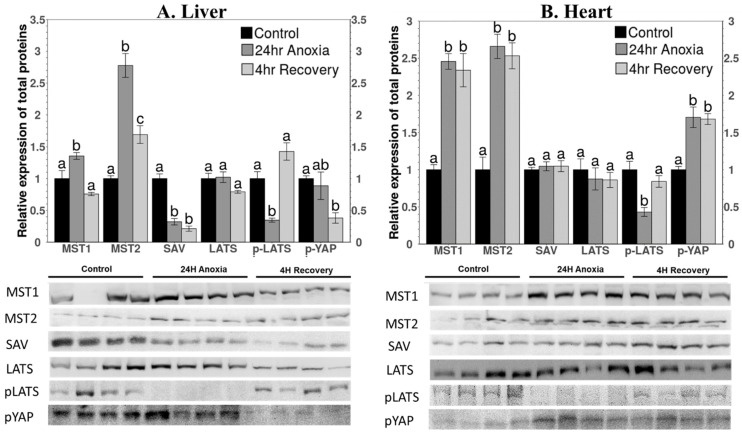
Relative expression levels of proteins involved in the Hippo pathway that are known to be cytoplasmic in (**A**) liver and (**B**) heart whole-tissue extracts of *R. sylvatica* under control, 24 h anoxia exposure, or 4 h aerobic recovery from anoxia. Proteins were detected by Western immunoblotting and immunoblot bands are shown below histograms. Data are mean ± SEM, *n* = 3–4 independent trials on samples from different animals. Data were analyzed using analysis of variance with a post hoc Tukey test; different letters denote values that are significantly different from each other (*p* < 0.05). Other information as Figures S1–S9.

Relative levels of proteins associated with the Hippo pathway that are known to be cytoplasmic were assessed in total protein extracts of liver and heart tissue from wood frogs and assessed using SDS-PAGE and immunoblotting. A significant increase in MST1 content was observed after 24 h anoxia exposure in both liver and heart, by 1.35 ± 0.05-fold and 2.45 ± 0.1-fold, respectively, compared to controls (Figure 2). However, during aerobic recovery (4 h back in normal air) the tissues differed in their responses. In liver, MST1 levels had decreased to 76 ± 2.5% of control values (not significantly different from controls) within 4 h (Figure 2A), whereas heart MST1 levels remained unchanged compared to anoxic values (Figure 2B).

The relative protein levels of MST2 increased strongly under anoxia in both liver (2.78 ± 0.18-fold) (Figure 2A) and heart (2.66 ± 0.16-fold) (Figure 2B) compared to controls. During recovery, MST2 responses were similar to those of MST1. MST2 levels in liver decreased significantly to a value intermediate between control and anoxic values (1.7 ± 0.14-fold over controls) (Figure 2A) but, again, MST2 values remained high in heart after 4 h aerobic recovery (Figure 2B).

The levels of SAV protein in liver decreased to just 32 ± 5% of controls in response to 24 h anoxia and had fallen further to 21.2 ± 4% of controls after 4 h aerobic recovery (Figure 2A). By contrast, no significant change was observed under either anoxia or recovery as compared to control values in heart (Figure 2B). No significant changes were observed in LATS protein levels in either tissue in response to anoxia or recovery (Figure 2). However, when the phosphorylation state of LATS protein was assessed, both tissues showed strong reductions in p-LATS content under anoxia, decreasing to 34.67 ± 3% and 43.14 ± 6% of the control values in liver and heart, respectively (Figure 2). However, phosphorylation of LATS returned to control values during aerobic recovery. Phosphorylation of YAP protein was also assessed; p-YAP is sequestered in the cytoplasm, preventing its translocation to nucleus to initiate gene expression. The responses by p-YAP differed between the two tissues. In liver, p-YAP content did not change during anoxia, but a significant decrease was observed during aerobic recovery to just 38 ± 8% of control levels (Figure 2A). In heart, the opposite effect was seen with a significant increase in p-YAP by 1.7 ± 0.13-fold during anoxia, compared to controls, and levels remaining unchanged after 4 h aerobic recovery (Figure 2B).

### 3.2. Protein Levels of Nuclear Components of the Hippo Pathway

**Figure 3:**
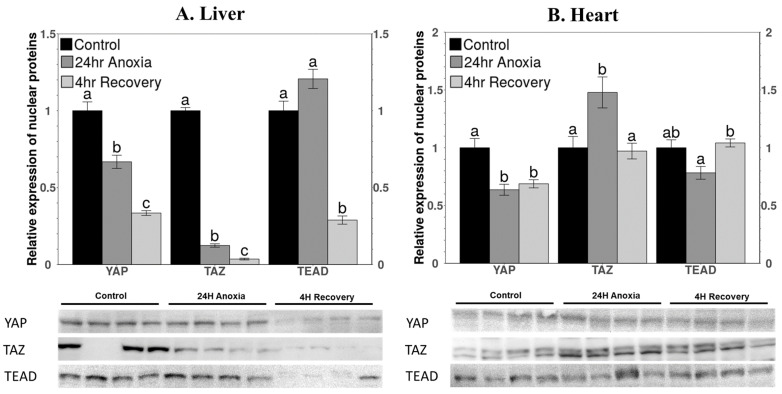
Relative expression levels of nuclear proteins (nuclear fraction) involved in the Hippo pathway in (**A**) liver and (**B**) heart of *R. sylvatica* under control, 24 h anoxia exposure, or 4 h aerobic recovery from anoxia conditions as determined by Western immunoblotting. Other information as in Figure 2.

Relative protein levels of three proteins belonging to the Hippo pathway that have primary functions in the nucleus (YAP, TAZ, TEAD) were evaluated in nuclear extracts of liver and heart tissue (Figure 3). In liver, YAP protein showed a significant decrease in the nuclear fraction during 24 h anoxia to 66.8 ± 4% of control values and fell further to 33.5 ± 1.5% of controls during aerobic recovery (Figure 3A). YAP levels in heart also decreased significantly during anoxia to 63.6 ± 4.8% of control values and remained unchanged during the 4 h recovery period (Figure 3B). The levels of TAZ in nuclear extracts decreased drastically to just 12.4 ± 1% in anoxic liver, as compared with controls, and fell further to just 3.6 ± 0.5% of controls during recovery (Figure 3A). By contrast, TAZ levels increased by 1.50 ± 0.13-fold over control values in anoxic heart, suggesting an important role for TAZ in anoxia heart (Figure 3B). However, TAZ levels were reduced again to control levels during aerobic recovery. TEAD protein levels were not significantly affected by 24 h anoxia in liver but had decreased to just 29 ± 2.7% of controls after 4 h aerobic recovery (Figure 3A). TEAD levels in heart showed little change between the three experimental groups, although a small but significant difference between anoxia and recovery conditions was detected (Figure 3B).

### 3.3. Protein Levels of OCT4 and SOX2 Downstream Targets

**Figure 4:**
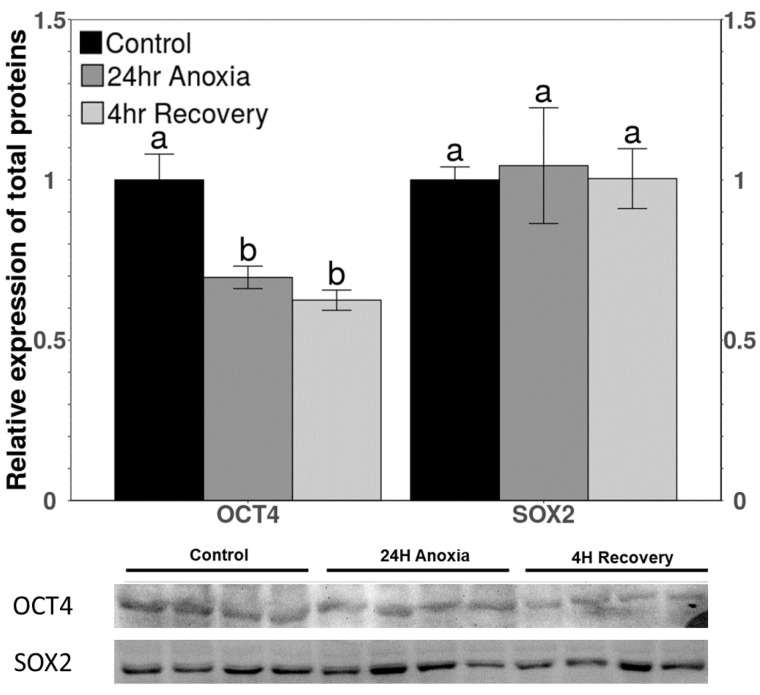
Relative expression levels of downstream targets of YAP/TEAD binding to promoter region in heart of *R. sylvatica* under control, 24 h anoxia exposure or 4 h aerobic recovery from anoxia conditions as determined by western immunoblotting. Other information similar to Figure 2.

The relative protein levels of OCT4 and SOX2 under control, anoxia, and aerobic recovery conditions in wood frogs were analyzed in liver in a previous study [20]. Figure 4 shows the comparable effects of these conditions on relative total protein levels of OCT4 and SOX2 in heart. Under anoxia, OCT4 levels decreased significantly to 69.5 ± 3.5% of control values and remained low during aerobic recovery. However, no significant change was observed in the relative levels of SOX2 protein among the three conditions.

### 3.4. DNA-Binding Activity of TEAD

**Figure 5:**
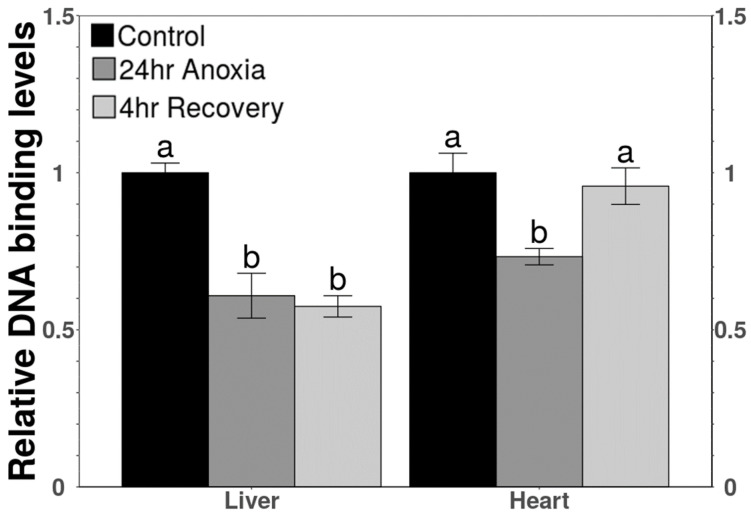
Relative DNA-binding levels of TEAD in total protein extracts in liver and heart of *R. sylvatica* under control, 24 h anoxia or 4 h recovery conditions as determined by TF-ELISA or DPI-ELISA. Other information similar to Figure 2.

The binding ability of TEAD to its consensus DNA sequence was examined using a transcription factor ELISA, previously known as DNA-protein binding ELISA (DPI-DNA protein interaction). A significant decrease was observed in DNA-binding levels by TEAD under anoxic conditions in both liver and heart. Binding capacity decreased to 60.8 ± 7% in anoxic liver and to 73.3 ± 2.6% in anoxic heart, as compared with controls. These levels remained unchanged during aerobic recovery in liver, but binding rose again to control levels after 4 h aerobic recovery in heart (Figure 5).

### 3.5. Transcript Levels of Key Components of the Pathway

**Figure 6:**
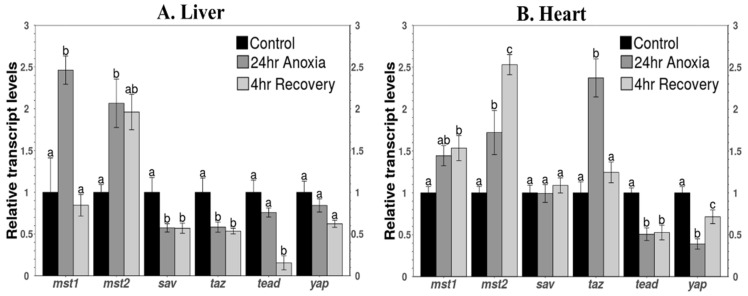
Relative expression of gene transcripts of the proteins involved in the Hippo pathway in (**A**) liver and (**B**) heart of *R. sylvatica* in control, 24 h anoxia, and 4 h recovery from anoxia as determined by qPCR. Other information similar to Figure 2.

Transcript levels of key genes involved in Hippo signaling were assessed in liver and heart (Figure 6). Transcript levels of mst1 increased significantly by 2.44 ± 0.12-fold under anoxia in liver but returned to control values during aerobic recovery (Figure 6A), whereas an increasing trend was observed in heart under anoxia with a significant increase of 1.5 ± 0.15-fold over controls during recovery (Figure 6B). Transcript levels of mst2 increased significantly during anoxia in both organs by 2.01 ± 0.29-fold and 1.72 ± 0.26-fold over controls in liver and heart, respectively (Figure 6). During recovery, mst2 transcripts remained unchanged in liver but increased further to 2.53 ± 0.12-fold over controls in heart.

Transcript levels of sav and taz decreased significantly during anoxia in liver to 57.4 ± 5% and 58.3 ± 6% of control values, respectively (Figure 6A) and remained unchanged during recovery. No significant changes were observed in the transcript levels of sav under all three conditions in heart, but taz transcripts increased significantly in heart under anoxia by 2.37 ± 0.23-fold but retuned to control values during recovery (Figure 6B). Transcript levels of tead showed a decreasing trend with no significant decrease in anoxic liver but a significant reduction during recovery to 15.5 ± 8.3% of controls (Figure 6A). In heart, the transcript levels of tead decreased significantly to 50.6 ± 7.5% of the control values, and the values remained unchanged during recovery (Figure 6B). Transcript levels of yap showed a decreasing trend but no significant change under any of the three conditions in liver, whereas yap transcript levels decreased significantly in anoxic heart to 39 ± 6% of controls but rose again to 71.4 ± 8.1% of control values during recovery in heart (Figure 6).

## 4. Discussion

Wood frogs can survive whole-body freezing over many months in the winter [1,2]. Stresses such as anoxia and ischemia arise because ice formation shuts down all movements, including breathing, blood circulation, and nerve transmission and isolates cells, tissues, and organs so that they must endure with only their own internal reserves and regulatory mechanisms to maintain viability. Under these conditions, tolerance of anoxia is essential. Indeed, frogs initiate and regulate the expression of many genes/proteins involved in multiple cellular processes in an energy-efficient manner to enable long-term survival without oxygen [38]. For example, the hypoxia-inducible factor (HIF-1) is undoubtedly one transcription factor that supports cell/tissue survival during freezing. The present study shows that the Hippo signaling pathway is involved in metabolic regulation under the limited availability of energy and oxygen to suppress gene transcription of the targets regulated by the YAP/TAZ/TEAD complex by preventing the translocation of YAP to the nucleus [9,15] to initiate stress-specific responses. Recent studies have also linked the Hippo pathway with ROS-mediated cellular responses or oxidative stress, where key components of the pathway act as ROS scavengers [39,40,41]. Taken together, it is evident that this pathway plays an important role in maintenance of overall cellular homeostasis under stress, and regulation study under anoxic conditions is pertinent.

The total protein levels of core components involved in the Hippo pathway were analyzed for liver and heart under control, 24 h anoxia, and 4 h recovery conditions. Protein levels of MST1 and MST2 were significantly elevated in both tissues during anoxia and after 4 h aerobic recovery from anoxia (Figure 2), and transcript levels of both *mst1* and *mst2* rose under anoxia (Figure 6). This indicates a transcription-factor-initiated process to upregulate genes whose transcribed mRNAs then support enhanced synthesis of selected proteins in response to anoxia. This makes sense given that MST1 and MST2 are the initial proteins of the Hippo pathway, and their elevation represents an activation of the pathway [42]. Further support for the importance of MST1/2 in initiating anoxia-triggered cell processes is supplied by the responses of SAV protein (the binding partner of MST1/2); both *sav* transcript and SAV protein levels were reduced (liver) or unchanged (heart) over anoxia/recovery.

A study by Zhou et al. using mouse hepatocytes showed that MST1 and MST2 negatively regulate the expression of YAP in mammalian liver [24]. The study further showed that MST1/2-deficient liver showed inhibition of the phosphorylation of YAP and therefore increased the abundance of YAP in the nucleus [24]. These findings are in accordance with the current study since a significant decrease in the protein levels of YAP were observed in nuclear fractions of both liver and heart. Moreover, transcript levels of *yap* were unchanged in the liver under anoxia, whereas levels decreased significantly in heart (Figure 3 and Figure 6). During recovery from anoxia, in liver, the protein levels decreased further, and the transcript levels of *yap* showed a decreasing trend with no significant difference, but in heart the YAP protein levels remained unchanged, and transcripts increased significantly during recovery (Figure 3 and Figure 6). Increased transcript levels represent preparation of the organ for protein translation in response to the upcoming event. During recovery, cardiomyocytes could experience a rush of oxygen into the blood that could potentially increase ROS levels [43,44]. An increased rate of ROS production during recovery causes oxidative stress and could lead to cardiomyocyte death. Activation of YAP could protect from cell death [41] through myocardial regeneration by promoting the proliferation of cardiomyocytes after myocardial injury, suggesting a protective role of YAP after oxidative stress [9,25].

A tissue-specific response was observed in the expression of SAV and p-YAP under both anoxia and aerobic recovery conditions, as compared to controls (Figure 2). Activation of the Hippo pathway initiates a series of kinase activities that would phosphorylate YAP and sequester it in the cytoplasm. YAP is known to be phosphorylated at multiple sites and inhibited by two mechanisms [45]. Phosphorylation at Ser-127 results in binding of 14-3-3 protein which mediates spatial regulation (cytoplasmic-nuclear shuttling) [46], whereas phosphorylation at Ser-381 is required for phosphodegron-induced protein degradation (i.e., primed for ubiquitination-based degradation) [45]. The current research focused on the analysis of relative levels of p-YAP at Ser-127, but a detailed study on multiple phosphorylation sites on YAP and AMPK levels during anoxia and recovery with respect to control for both the tissues could be used to further validate the findings presented in this paper.

In the current study, expected results with respect to activation of the Hippo pathway were observed in the heart. The levels of p-YAP increased during anoxia and remained unchanged during recovery; however, the levels of SAV and LATS showed no significant change in any condition. Interestingly, liver showed contrasting results for the relative protein expression of SAV and p-YAP. A significant decrease in the levels of SAV, as well as p-YAP, was observed (Figure 2). Multiple studies have reported that activation of MST1/2 under stress is sufficient to turn off YAP/TAZ [23,24,47,48] by directly phosphorylating MST1/2. It was also observed that under energy stress, AMPK directly phosphorylates YAP to prevent its interaction with TEAD and inhibiting YAP/TAZ/TEAD-mediated gene transcription [15,49]. Previous studies have described an organ-specific increase in AMPK levels in response to environmental stress in stress-tolerant animals [50,51,52,53,54].

Therefore, multiple possibilities could be inferred: (1) the increased levels of AMPK and MST1/2 could lead to direct phosphorylation of YAP in the heart without other components of the Hippo pathway being involved; (2) in heart, YAP is sequestered in the cytoplasm by phosphorylation at Ser-127 so it can be immediately dephosphorylated and translocated to the nucleus to initiate cytoprotective gene response when oxidative stress is encountered; (3) in liver, YAP is phosphorylated at S-381 to be tagged for immediate degradation, thereby reducing YAP/pYAP levels; (4) posttranslational modifications of YAP such as GlcNAcylation and ubiquitination could also be responsible for the varied expression of YAP. However, the specific regulation pattern remains elusive and therefore, further research in this context is required.

The DNA-binding levels of TEAD to the promoter regions of gene sequences of its downstream targets during anoxia decreased significantly during 24 h anoxia for both tissues and remained unchanged in liver after aerobic recovery but increased to return the control levels in the heart after 4 h recovery (Figure 5). This observation is in accordance with the relative expression of proteins in nuclear fractions of the liver and heart for all the stresses, showing a similar trend (Figure 3). The transcript levels of *tead* showed a decreasing trend compared to controls in the anoxic liver that was further reduced during recovery (Figure 6A). Decreased transcript and total protein levels during recovery could be linked with reduced binding in the liver. The decreased levels of YAP and TAZ that act as a co-activators of TEAD (to enhance its binding to the promoter region of target genes), further justifies the decreased DNA binding [55,56]. Cells encountering anoxic conditions reduce their metabolic rate to decrease ATP consumption. They reprioritise the expenditure of available energy for cell survival pathways by regulating the expression of selected transcription factors that include OCT4 and SOX2 [16,17]. In previous study from our lab, similar changes in total protein levels and DNA-binding ability of OCT4 were observed under anoxia conditions in wood frog liver [20].

The binding of TEAD increased during recovery in heart for selective activation of genes (Figure 5). The increased binding levels coincide with increased total protein levels of TEAD during recovery from anoxia in wood frog heart (Figure 3B). Selective gene activation could be assumed since a different trend was observed in the total protein levels of OCT4 and SOX2, which are amongst the important downstream targets of YAP/TAZ mediated TEAD activation. It was observed that in heart, total protein levels of OCT4 decreased significantly during anoxia and remained low during recovery whereas the levels of SOX2 did not change under any condition compared to control (Figure 4). The relative levels of OCT4 and SOX2 during anoxia and recovery were studied in liver previously in our lab [20]. The study showed no significant change in the levels of OCT4 during anoxia as well as recovery but levels of SOX2 increased significantly during anoxia and further during recovery as compared to controls [20]. SOX2 is responsible for the regulation of cell survival pathways in response to low oxygen levels [57,58]. In addition, SOX2 is also involved in tissue repair and maintaining self-renewal capacity [59,60]. Consistent or increased expression levels of SOX2 (in a tissue-specific manner) represent the possibility that it plays an essential role in tissue regeneration and repair in response to increased ROS during recovery from anoxia (or other stresses that restrict oxygen availability such as freezing).

Surprisingly, a significant increase was observed in the protein levels of TAZ in the anoxic heart, but levels returned to control values during aerobic recovery. By contrast, TAZ levels decreased significantly in anoxic liver (Figure 3). The transcript levels of *taz* showed a similar pattern for both tissues (Figure 6). Reduced levels of TAZ in the nucleus in response to anoxia in the liver is consistent with activation of the Hippo signaling pathway since YAP/TAZ is sequestered via phosphorylation. TAZ has been characterized as a transcriptional coactivator for Runx2, PPARγ, TEAD, T-Box transcription factors, and SMAD complexes [61,62,63,64]. It is suggested to function as a transcriptional modulator and regulator of cellular functions. TAZ is also known to act as an enhancer to MyoD in response to injuries of adult muscle (skeletal, smooth, and cardiac) [65] by directly interacting with MyoD in the nucleus to accelerate its DNA-binding activity. MyoD is required for the repair and regeneration of muscle fibers [66,67]. Prolonged oxygen deficiency or anoxic conditions could lead to detrimental effects where heart muscle could tend to lose contractility [68,69]. Prolonged exposure to such conditions might cause atrophy [70,71,72]. Therefore, increased TAZ in the heart could potentially activate/enhance the function of MyoD to initiate repair mechanisms during/after anoxia stress.

Hence, it can be deduced that, under anoxic conditions, the small yet significantly increased levels of major cytoplasmic components and decreased levels of nuclear components represent the possibility of activation of the Hippo pathway. Activation of the pathway phosphorylates and sequesters YAP in the cytoplasm. Phosphorylated YAP restrains the translocation of YAP/TAZ in the nucleus, preventing the formation of the YAP-TAZ-TEAD complex that could have bound to the promoter region of selective genes and activated the gene expression. Increased transcript and protein levels of TAZ are essential to protect cardiac muscle during anoxia. The unchanged/increased total protein levels of SOX2 in heart and liver during anoxia and recovery could activate tissue repair under increased oxidative stress. In total, it is proposed that the Hippo pathway is activated to suppress gene expression under anoxic conditions, allowing for energy conservation and increasing the chances of survival under oxygen-restricted conditions but further research in this aspect is warranted.

## Data Availability

The data that support the findings of this study are available from the corresponding author upon reasonable request.

## Supplementary Materials

The following are available online at https://www.mdpi.com/article/10.3390/life11121422/s1, Figure S1. The ECL showing total protein levels of MST1 for.(A) liver and (B) heart whole tissue extracts of R. sylvatica. L ane 1 representing a protein mol. wt. ladder, lane2 to 5 represents control samples, lane 6 to 9 represents 24 h anoxia exposure and lane 10 13 represent 4 h aerobic recovery from anoxia. The protein quantified is highlighted in red; Figure S2. The ECL showing total protein levels of MST2 for (A) liver and (B) heart whole-tissue extracts of R. sylvatica. Other information similar to Figure S1; Figure S3. The ECL showing total protein levels of SAV for (A) liver and (B) heart whole-tissue extracts of R. sylvatica. Other information similar to Figure S1; Figure S4. The ECL showing total protein levels of LATS for (A) liver and (B) heart whole-tissue extracts of R. sylvatica. Other information similar to Figure S1; Figure S5. The ECL showing total protein levels of pLATS for (A) liver and (B) heart whole-tissue extracts of R. sylvatica. Other information similar to Figure S1; Figure S6. The ECL showing total protein levels of pYAP for (A) liver and (B) heart whole-tissue extracts of R. sylvatica. Other information similar to Figure S1; Figure S7. The ECL showing total protein levels of YAP for (A) liver and (B) heart whole-tissue extracts of R. sylvatica. Other information similar to Figure S1; Figure S8. The ECL showing total protein levels of TAZ for (A) liver and (B) heart whole-tissue extracts of R. sylvatica. Other information similar to Figure S1; Figure S9. The ECL showing total protein levels of TEAD for (A) liver and (B) heart whole-tissue extracts of R. sylvatica. Other information similar to Figure S1.
