# Supplementary material for: Activation of the Hippo Pathway in Rana sylvatica: Yapping Stops in Response to Anoxia
Source: Life (Basel). 2021 Dec 17;11(12):1422. doi: 10.3390/life11121422 (PMC8708225; doi:10.3390/life11121422)
Supplement: Supplementary file 1 [file life-11-01422-s001.zip › life-1458793-supplementary.pdf]

life

Supplementary Material of Activation of the Hippo Pathway in *Rana sylvatica*: Yapping Stops in Response to Anoxia

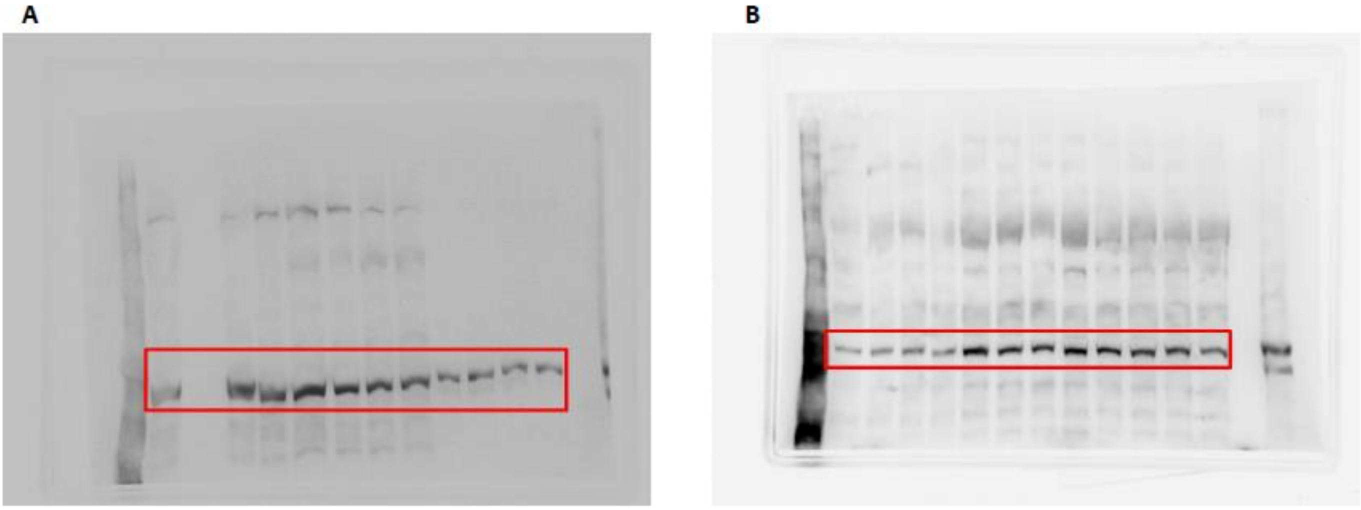

Figure S1. The ECL showing total protein levels of MST1 for.(**A**) liver and (**B**) heart whole tissue extracts of R. sylvatica. L ane 1 representing a protein mol. wt. ladder, lane2 to 5 represents control samples, lane 6 to 9 represents 24 h anoxia exposure and lane 10 13 represent 4 h aerobic recovery from anoxia . The protein quantified is highlighted in red.

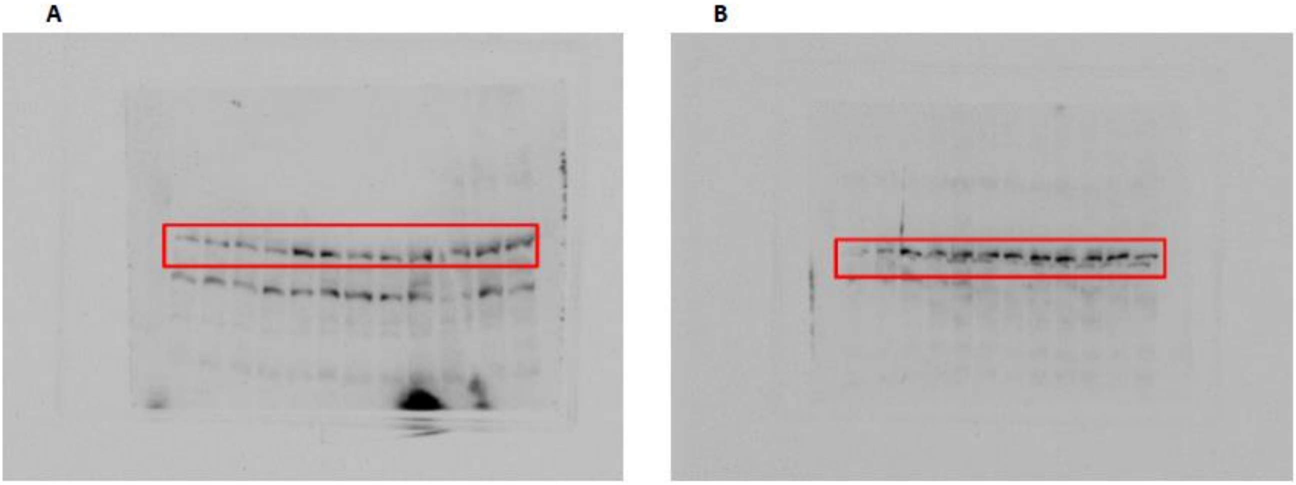

Figure S2. The ECL showing total protein levels of MST2 for (**A**) liver and (**B**) heart whole-tissue extracts of R. sylvatica. Other information similar to Figure S1.

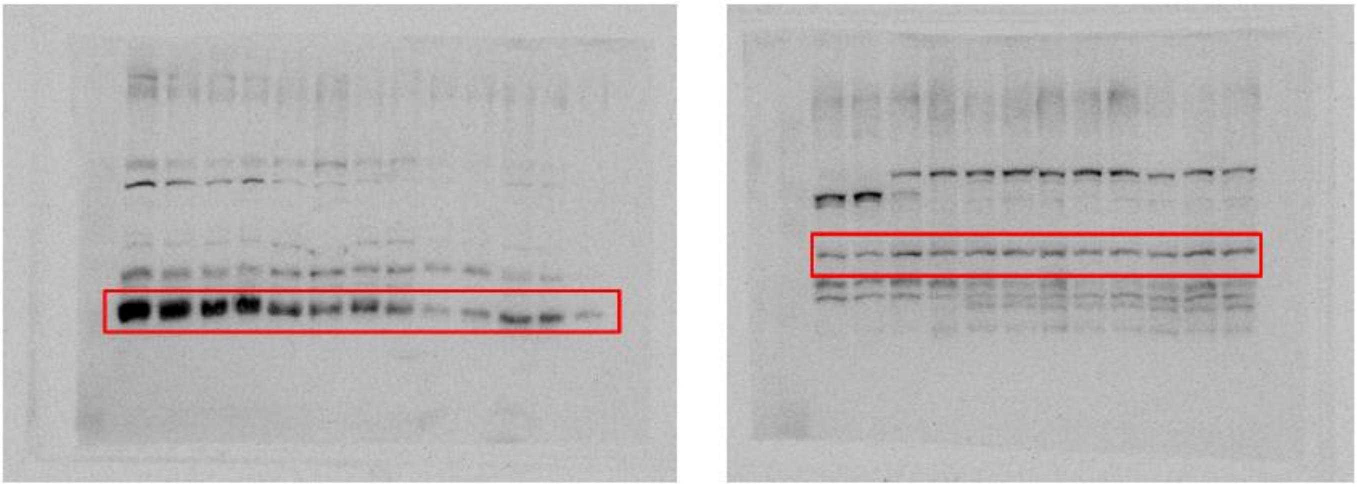

Figure S3. The ECL showing total protein levels of SAV for (**A**) liver and (**B**) heart whole-tissue extracts of R. sylvatica. Other information similar to Figure S1.

A B

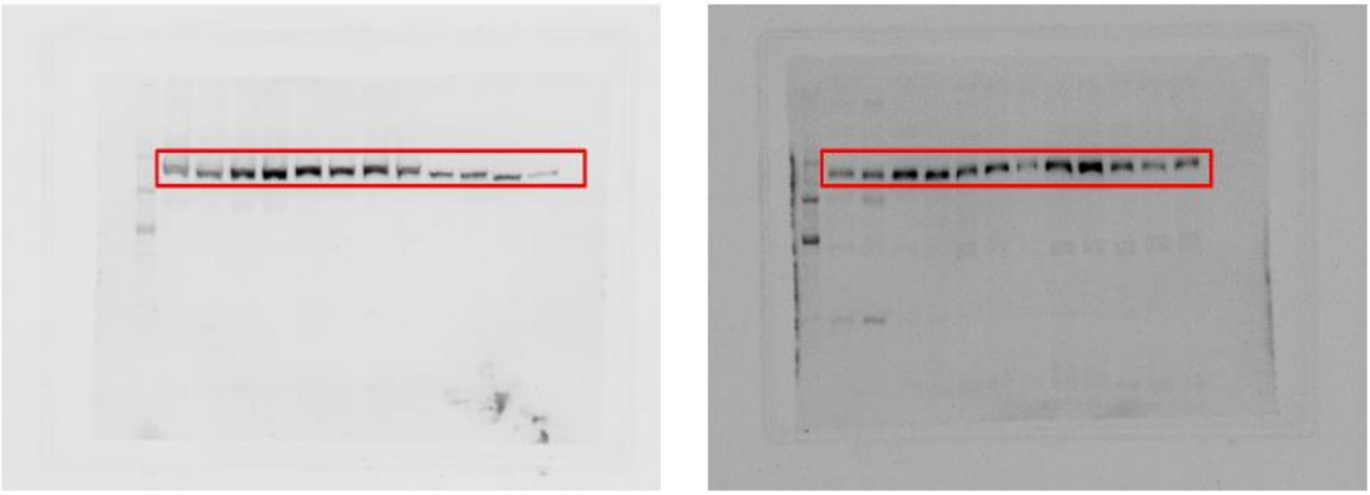

Figure S4. The ECL showing total protein levels of LATS for (**A**) liver and (**B**) heart whole-tissue extracts of R. sylvatica. Other information similar to Figure S1.

A B

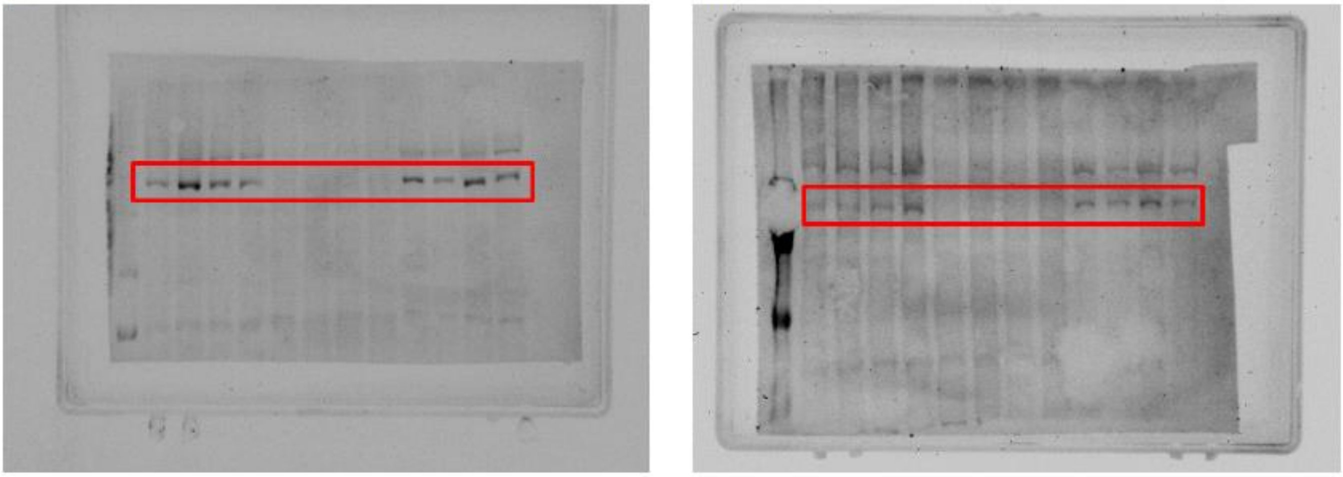

Figure S5. The ECL showing total protein levels of pLATS for (**A**) liver and (**B**) heart whole-tissue extracts of R. sylvatica. Other information similar to Figure S1.

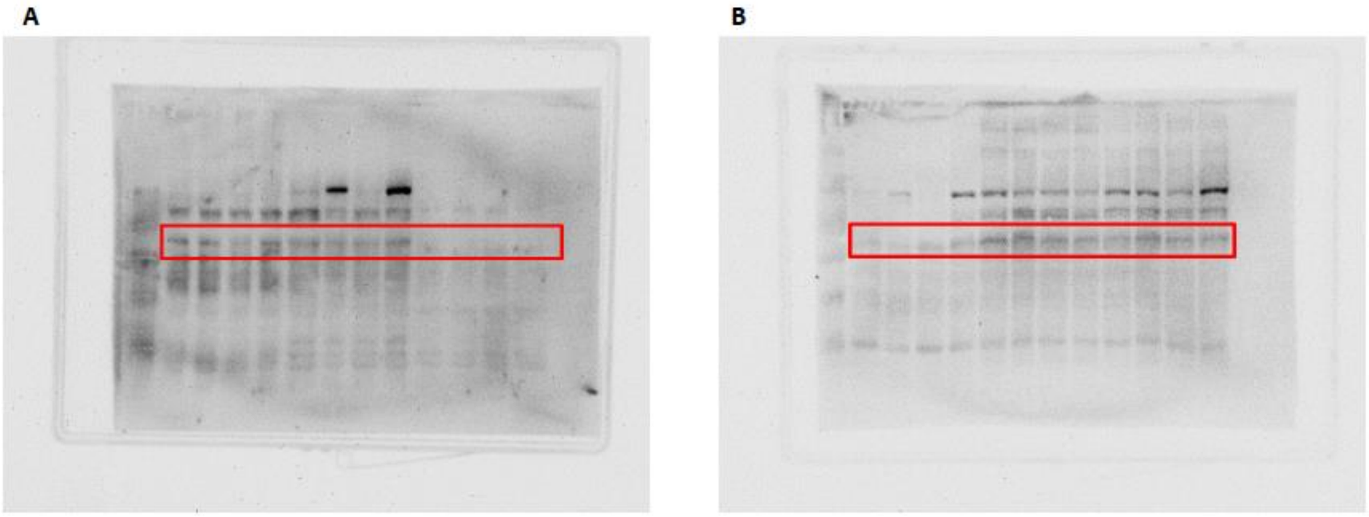

Figure S6. The ECL showing total protein levels of pYAP for (**A**) liver and (**B**) heart whole-tissue extracts of R. sylvatica. Other information similar to Figure S1.

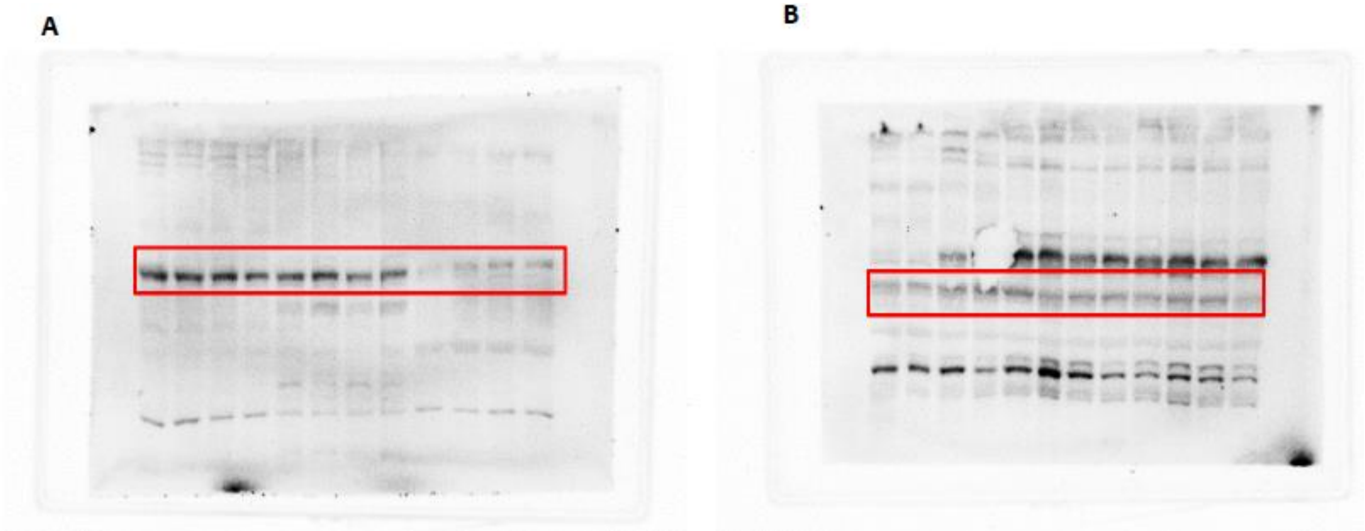

Figure S7. The ECL showing total protein levels of YAP for (**A**) liver and (**B**) heart whole-tissue extracts of R. sylvatica. Other information similar to Figure S1.

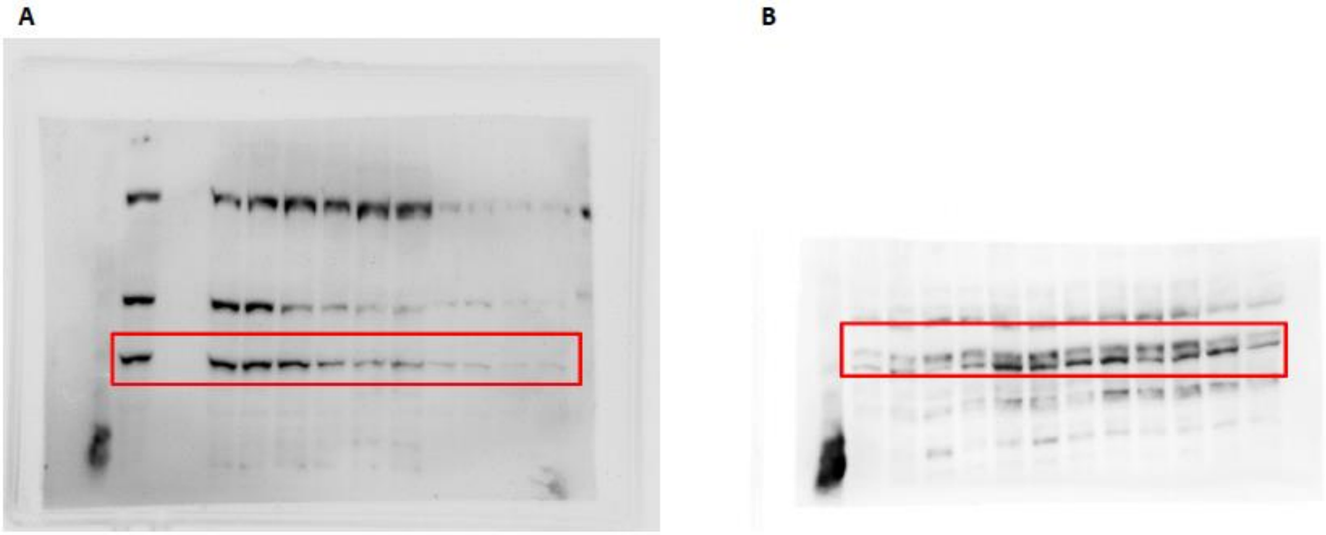

Figure S8. The ECL showing total protein levels of TAZ for (**A**) liver and (**B**) heart whole-tissue extracts of R. sylvatica. Other information similar to Figure S1.

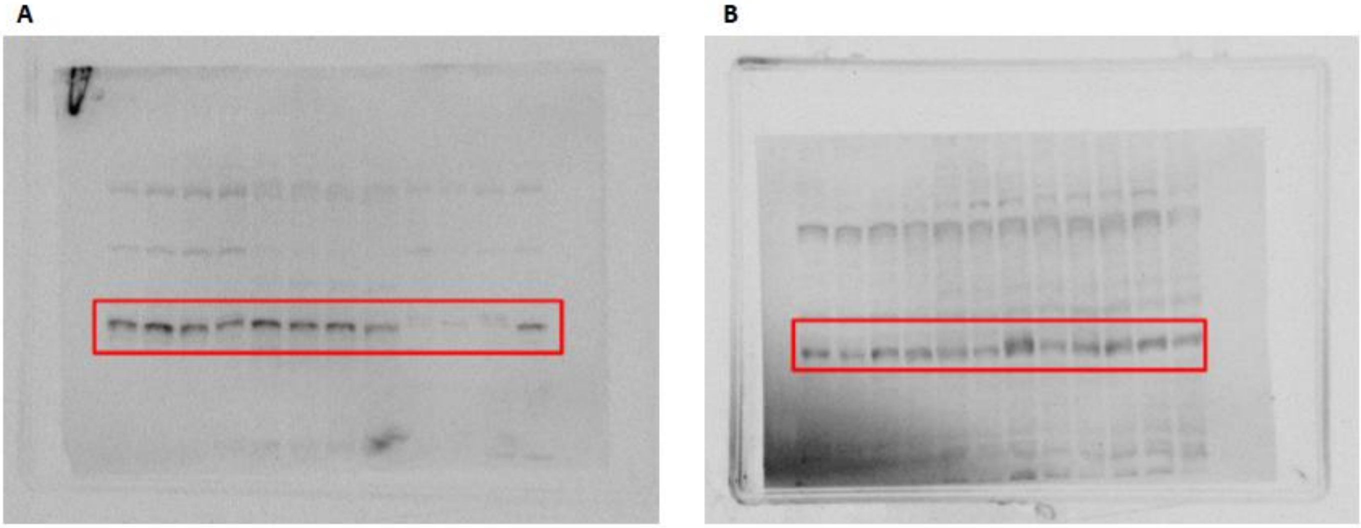

Figure S9. The ECL showing total protein levels of TEAD for (**A**) liver and (**B**) heart whole-tissue extracts of R. sylvatica. Other information similar to Figure S1.
